# 

**Published:** 1880-07

**Authors:** 


### Books and Periodicals.

NUGGETS OF GOLD is the title of a neat practical book on the laws of success in Life. The author is Mr. John Heermans, of Corning, who is no novice as a writer. Mr. Heermans has been a prolific contributor to our local press and invariably has had something to say and always has said it well. His book embodies the mature reflections of a man who has looked at society with both eyes wide open. We commend the book to the young men who may desire to profit by the experiences of a mature and thoughtful one. We do not agree with Mr. Heermans in his denunciation of liquor for "medicinal purposes," but no one can go wrong to accept his conclusious upon the liquor question.

THE SEA WORLD AND FISHING GAZETTE.—The angling fraternity will be glad to learn that Charles Hallock, so long editor of *Forest and Stream* has become editor and proprietor of a strictly fisherman's paper, bearing the above title. It needs no further commendation than to announce Mr. Hallock as editor. $2 per year, address 113 Fulton St., New York.

THE CHICAGO MEDICAL GAZETTE.—It is a genuine pleasure to read a medical journal bearing the stamp of originality upon every page. Such a journal is the one above named. It is edited by Dr. E. C. Dudley, who is aided by a corps of eight assistants, every one of whom is an authority in the special department of the journal under his care. For crisp, original and practical reading the *Chicago Med. Gazette* is unsurpassed. Published on the 5th and 20th of each month at $2.00 per year. Address:—Chicago, Ills.

THE THERAPEUTIC GAZETTE.—This is a well printed, comely magazine, ably conducted by Dr. Wm. Bodie. It is devoted to the study and introduction of new therapeutic agents, occupying an exclusive field. It is of real use to every practicing physician. Monthly, $1.00 per year. Address, Geo. J. Davis, Medical Publisher, Detroit, Mich.

WALSH'S RETROSPECT—a quarterly compendium of American Medicine and Surgery. Edited by Ralph Walsh, M. D., and Thos. E. McArdle, M. D., at Washington, D. C. This is an octavo magazine of 150 pages, well edited, illustrated, and printed upon fine paper. $2.00 a year—worth twice the sum.

THE MEDICAL NEWS AND ABSTRACT.—Edited by J. Minis Hayes, A. M., M. D., at Philadelphia. Octavo, magazine form, over 64 pages. Monthly, $2.50 a year. A very cheap and useful journal for every medical man.

**TERMS.—50 Cents a Year. Address The Bistoury, Elmira, N. Y.**

### SUBSCRIPTIONS RECEIVED TO JULY, 1880

In this column we will credit every subscription received during the last quarter. Subscribers will please notify us immediately if their payments are not duly credited. The State only is given—this, with the full name of the party subscribing, will be sufficient to indicate who is meant.

NEW YORK—Geo H Greenway, J J Keyes, Dr Hubbard, Thos Johnson, Dr L. Swiss, J H Barry, Dr E Jones, J L Cooley $1.00, Miss Clara A Spaulding, Mrs Chas Rapelyea, Wm Collins, Dr Dan Dunn, G H Platt $1.00, Dr Fox, Reed and Conwich, Dr L Looms, Dr D M Taylor, Dr Geo Smith, Dr Lydenham, Dr J Look, Dr Frederick Jaskel, Dr D White $4.00, Prof A N Norton, Miss Fannie Miller, L. I.

PENNSYLVANIA—Dr Pevcival Herman, Dr A Funk. Dr A K Minich $1.00, Dr Long, Dr R R Chelson, Dr D D Loop, John Dixey, Dr Geo Wolf, Dr M L Davis $1.00, Dr I B Smith, The Underwriter, Dr Ben Harris, Dr John Y Shindel, Dr. Dodge, Robert Snodgrass $1.00, Dr Thos J. Birch, Dr Geo Krouse, Robt Thomas, Dr F Schnure.

DISTRICT OF COLUMBIA—Mrs John J Baldwin $1.00, A Zoller, A B Martin, Surgeon General's Office $1.00.

OHIO—Dr John Suseman, Dr N Bloomer,

PHILLIPPINE ISLAND—F J. Stebbins.

U. S. ARMY—Dr J H Baxter, Dr S Bliss.

SOUTH CAROLINA—Butcley Grunball $1.00, Dr Geo English, Dr P Jarvis.

VERMONT—L D Ross $1.00, Dr Snow, Dr O B Howe, Dr Jane Crouse.

INDIANA—Rosalie A Fisher $2.50, Dr Wm Faulkner, Dr Storms.

KANSAS—Mrs Jas D Brown.

MICHIGAN—Chas Bassett.

MARYLAND—Jos P Elliott $2.00, Dr H Coons, Dr Millhans.

MISSOURI—Dr J S Wallace, Dr P Griffin, Dr O Brown, Electic Med. Journal.

NEBRASKA—Dr R B Wallace, Dr Hunt.

